# Long-term prognosis of short QT interval in Korean patients: a multicenter retrospective cohort study

**DOI:** 10.1186/s12872-020-01824-3

**Published:** 2021-01-06

**Authors:** Dae-Young Kim, Jae-Sun Uhm, Min Kim, In-Soo Kim, Moo-Nyun Jin, Hee Tae Yu, Tae-Hoon Kim, Jong-Youn Kim, Boyoung Joung, Hui-Nam Pak, Moon-Hyoung Lee

**Affiliations:** 1 Division of Cardiology, Department of Internal Medicine, Severance Cardiovascular Hospital, Yonsei University College of Medicine, 50-1 Yonsei-ro Seodaemun-gu, Seoul, 03722 Republic of Korea; 2 Division of Cardiology, Department of Internal Medicine, Yongin Severance Hospital, Yonsei University College of Medicine, Yongin-si, Gyunggi-do Republic of Korea; 3 Division of Cardiology, Department of Internal Medicine, Sanggye Paik Hospital, Inje University College of Medicine, Seoul, Republic of Korea; 4 Division of Cardiology, Department of Internal Medicine, Gangnam Severance Hospital, Yonsei University College of Medicine, Seoul, Republic of Korea

**Keywords:** Atrial fibrillation, QT interval, Short QT syndrome, Sudden cardiac arrest, Ventricular arrhythmia

## Abstract

**Background:**

Short QT syndrome is a rare, inherited channelopathy associated with sudden cardiac arrest (SCA) but the characteristics and prognosis of short QT interval (SQTI) in Korean patients remain unclear. This study aimed to determine the clinical characteristics and outcomes of SQTI in a Korean population.

**Methods:**

Consecutive patients with SQTI from January 1999 to March 2019 in three university hospitals in South Korea were recruited. SQTI was defined as a Bazett’s formula-corrected QT interval (QTc) ≤ 340 ms in serial electrocardiograms. Age- and sex-matched patients with a normal QTc and without overt cardiovascular disease were included in a 1:4 ratio. Clinical and ECG features and outcomes were compared between patients with and without SQTI.

**Results:**

34 patients with SQTI [age, 23.5 (21–30.5) years; 31 male] were followed up for 4.8 (2.0–7.8) years. Early repolarization, tall T wave, and U wave were significantly more frequent in patients with SQTI than in those without SQTI. QT dispersion [44.0 (28.0–73.0) vs. 20.0 (12.0–35.0) ms, *P* < 0.001] was significantly wider and heart rate [52.0 (47.0–58.0) vs. 70.0 (62.3–84.0)/min, *P* < 0.001] was significantly slower in patients with SQTI than in those without. Atrial fibrillation (AF, 11.8% vs. 2.2%, *P* = 0.030) and ventricular arrhythmia (VA)/SCA (8.7% vs. 0%, *P* = 0.007) were significantly more frequent in patients with SQTI than in those without. SQTI was significantly associated with AF [odds ratio, 5.911; 95% confidence interval, 1.257–27.808; *P* = 0.025] and VA/SCA.

**Conclusions:**

In this subset of Korean population, SQTI was associated with AF and VA/SCA.

## Background

Short QT syndrome (SQTS) is a rare, life-threatening, inherited channelopathy associated with ventricular arrhythmia (VA) and sudden cardiac arrest (SCA) [1]. The first reported clinical case was of familial short QT interval < 300 ms accompanied by arrhythmia [2]. After a while, familial sudden cardiac death (SCD) accompanied with short QT interval and a history of syncope or palpitations was reported [3]. Later, SQTS was found to increase the risk of SCD, especially in young patients who were not previously diagnosed with any disease. The clinical and electrocardiogram (ECG) features, as well as clinical outcomes, remain unclear in Korean patients with SQTS. This is due to the rarity of the condition and the relatively short time since its discovery in Western countries. The aim of our study was to determine the clinical characteristics and outcomes of patients with short QT interval (SQTI) in a Korean population.

## Methods

### Study population

This was a multicenter retrospective cohort study. The study design was approved by the Institutional Review Board of Yonsei University Health System (IRB approval number: 4-2019-0644), and the study was conducted in accordance with the Declaration of Helsinki. The institutional review board waived both the need for the acquisition of informed consent from patients to be included in the analysis and the need for review by a critical event committee owing to the study’s retrospective nature and the absence of patient identification data.

**Fig. 1:**
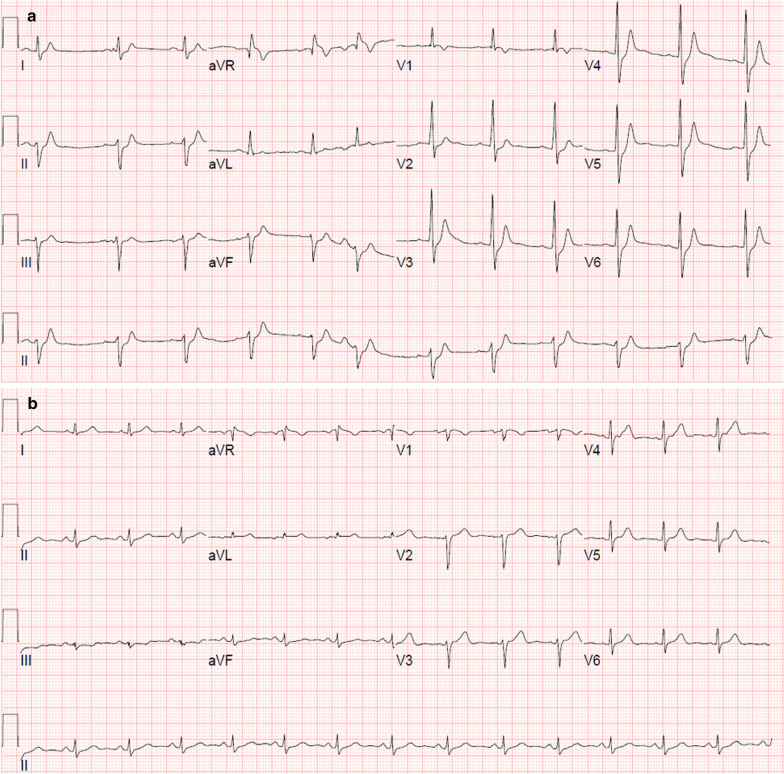
Representative twelve-lead ECG. A) A patient with short QT syndrome who experienced sudden cardiac arrest. QT interval, 306 ms; corrected QT interval, 330 ms; QRS duration, 88 ms; and and heart rate, 70/min. B) A patient with normal QTc without any clinical history who visited the outpatient clinic due to upper respiratory infection. QT interval, 366 ms; corrected QT interval, 432 ms; QRS duration, 94 ms; and heart rate, 84/min

We included consecutive patients with SQTI from January 1999 to March 2019 in three university hospitals in South Korea. SQTI was defined as a Bazett’s formula-corrected QT interval (QTc) ≤ 340 ms in serial ECGs regardless of symptoms or history of familial SCD. For estimating the QT interval, we used GE Healthcare's 12SL software (Milwaukee, WI, USA). A representative beat was generated from the selected segment of each lead, and then a composite beat was formed by the representative beats of all independent leads. The global QT interval was considered as the longest QT interval in the multiple leads. Patients of all ages who met the SQTI definition were included in the SQTI group, even in cases without related symptoms or a history of familial SCD (Fig. 1). The Exclusion criteria were as follows: temporary SQTI due to electrolyte imbalance (e.g. hypercalcemia, hyperkalemia), acidosis, hyperthermia, use of digitalis and cholinergic drugs which were related to clinical impairments, especially during hospitalization; structural heart disease with ischemic or nonischemic cardiomyopathy; and significant (high-degree and third-degree) atrioventricular block (AVB). Age- and sex-matched subjects in the normal QT group were recruited in a 1:4 ratio and had at least three ECGs with normal QTc which were performed from January 1999 to March 2019 in the outpatient clinic. Patients with overt cardiovascular disease, defined as cardiomyopathy, coronary artery disease, and significant bradyarrhythmia, were excluded. Clinical outcomes included arrhythmia outcomes and all-cause death. Arrhythmia outcomes included atrial fibrillation (AF), VA/SCA, and Mobitz type 2 s-, high-, and third-degree AVB.

### Data analysis

The following clinical variables were collected from all recruited subjects: age, sex, height, weight, systolic and diastolic blood pressure, clinical history of loss of consciousness, palpitations, chest pain, SCA, the list of medications, familial history of SCA, accompanying arrhythmias (AF, VA, premature ventricular complex, supraventricular tachycardia, first-degree AVB, second-degree AVB, high-degree AVB, third-degree AVB), and ECG parameters (PR interval, QT interval, QT dispersion, heart rate, JT interval, biphasic negative–positive T wave, early repolarization, and tall T waves). Tall T waves were defined as > 10 mm (1 mV) in any of the precordial leads.[4] QT dispersion was calculated as the difference between the maximum and minimum QT interval in the 12-lead ECG. JT interval was measured from the J point to the T wave peak. Early repolarization was defined as an elevation of the QRS–ST junction (J point) in leads other than V1–V3.[5]

We compared the baseline characteristics including symptoms, family history, ECG findings, and clinical outcomes between the two groups.

### Statistical analysis

The results are presented as median (interquartile range) for continuous data and as frequency (percentage) for categorical data. To compare the clinical parameters between groups, we used the Mann Whitney U-test for continuous data and Fisher’s exact test for categorical data since all data sets were non-normally distributed. Univariate and multivariate logistic regression analyses were performed to determine the odds ratio of arrhythmia in patients with SQTI. A p-value ≤ 0.05 was considered statistically significant. Data were analyzed using Statistical Package for the Social Sciences version 25.0 (IBM Corporation, Armonk, NY).

## Results

A total of 4,694,382 ECGs were performed in 2,083,360 patients (both inpatients and outpatients) during the study period; 34 patients with SQTI were found, corresponding to a prevalence of 0.001%. These 34 patients [age, 23.5 (21.0–30.5) years; 31 male] with SQTI were followed up for 4.9 (2.0–7.8) years. Table 1 shows the baseline characteristics, medications, symptoms, and ECG characteristics of patients in the SQTI and normal QT groups. In the SQTI group, males younger than 40 years were dominant (91.2%). The medications in the SQTI and normal QT groups were not significantly different except for digoxin (5.9% vs. 0.7%, *P* = 0.041); drugs that were known to shorten QT interval were not administered to patients in the SQTI and normal QT groups. Among the 34 patients with SQTI, 19 patients (55.9%) visited the hospitals for the evaluation of symptoms such as chest pain, chest discomfort, dyspnea, palpitations, and dizziness. The others had various causes of visiting the hospitals [hospitalization for other causes (n = 8, 23.5%), routine health check-up (n = 4, 11.8%), preoperative evaluation for non-cardiac surgery (n = 3, 8.8%) in order of frequency]. Symptoms included palpitations (n = 4, 11.8%), loss of consciousness (n = 3, 8.8%), and chest pain (n = 3, 8.8%). Palpitations (11.8% vs. 2.2%, *P* = 0.030) were significantly more frequent in the SQTI group than in the normal QT group. Regarding ECG findings, QT dispersion was significantly wider and heart rate was significantly slower in the SQTI group than in the normal QT group. Early repolarization (n = 25, 73.5%), tall T waves (n = 20, 58.8%), and U waves (n = 4, 11.8%) were significantly more frequent in the SQTI group than in the normal QT group. There were no significant differences in the frequencies of premature ventricular contraction and supraventricular tachycardia between groups. Among patients with SQTI, twelve patients underwent transthoracic echocardiography during the follow-up period, which revealed no structural cardiac abnormalities.

**Table 1:** Baseline characteristics of the SQTI and normal QT groups

	SQTI group (n = 34)	Normal QT group (n = 136)	*P*
Demographics			
Age (year)	23.5 (21.0–30.5)	23.5 (21.0–31.0)	> 0.999
Male	31 (91.2)	124 (91.2)	> 0.999
Family history of SCA	1 (2.9)	0 (0.0)	0.200
Height (cm)	173.0 (170.0–176.8)	173.0 (168.0–177.0)	0.698
Weight (kg)	66.2 (61.1–72.0)	69.5 (60.0–78.8)	0.155
Systolic BP (mmHg)	124.0 (113.3–129.0)	122.0 (113.3–134.0)	0.537
Diastolic BP (mmHg)	71.0 (66.0–78.0)	76.0 (67.3–83.0)	0.056
ICD implantation	1 (2.9)	0 (0.0)	0.200
Medications			
Digoxin	2 (5.9)	1 (0.7)	0.041
β-blocker	3 (8.8)	7 (5.1)	0.415
Calcium channel blocker	5 (14.7)	7 (5.1)	0.052
RASi	3 (8.8)	6 (4.4)	0.304
Antiarrhythmic drug	2 (5.9)	2 (1.5)	0.129
Amiodarone	1 (2.9)	1 (0.7)	
Flecainide	1 (2.9)	1 (0.7)	
Diuretics	3 (8.8)	7 (5.1)	0.415
Lipid lowering agent	1 (2.9)	2 (1.5)	0.560
Antiplatelet	1 (2.9)	3 (2.2)	0.800
Anticoagulant	1 (2.9)	5 (3.7)	0.835
Symptoms			
Loss of consciousness	3 (8.8)	4 (2.9)	0.144
Palpitations	4 (11.8)	3 (2.2)	0.030
Chest pain	3 (8.8)	8 (5.9)	0.461
ECG characteristics			
Heart rate (/min)	52.0 (47.0–58.0)	70.0 (62.3–84.0)	< 0.001
PR interval (ms)	160.0 (146.0–175.5)	152.0 (137.5–168.0)	0.061
QT interval (ms)	361.0 (335.0–375.5)	380.0 (358.0–400.0)	0.001
QTc (ms)	334.0 (330.3–337.0)	416.0 (396.3–436.8)	< 0.001
QT dispersion (ms)	44.0 (28.0–73.0)	20.0 (12.0–35.0)	< 0.001
JT interval (ms)	262.0 (245.0–279.0)	200.0 (169.0–224.0)	< 0.001
U wave	4 (11.8)	0 (0)	–^a^
Biphasic T wave	1 (2.9)	0 (0)	–^a^
Early repolarization	25 (73.5)	14 (10.3)	< 0.001
Tall T waves	20 (58.8)	10 (7.4)	< 0.001
PVC	2 (5.8)	2 (1.5)	0.179
SVT	2 (5.8)	2 (1.5)	0.179
Follow-up period (year)	4.9 (2.0–7.8)	3.8 (3.8–4.2)	0.318

The results are presented as median (interquartile range) for continuous data and as frequency (percentage) for categorical data

*BP* blood pressure, *ICD* implantable cardioverter-defibrillator, *RASi* renin angiotensin system inhibitor, *PVC* premature ventricular contraction, *SQTI* short QT interval, *SVT* supraventricular tachycardia

^a^Statistical comparison could not be performed because the number of patients was small

Table 2 shows the arrhythmia outcomes. AF (11.8% vs. 2.2%, *P* = 0.030) was significantly more frequent in the SQTI group than in the normal QT group. Three patients experienced VA/SCA in the SQTI group, whereas no patients experienced VA/SCA in the normal QT group (8.7% vs. 0%, *P* = 0.007). One patient had ventricular fibrillation and underwent cardioverter-defibrillator implantation (Fig. 1a). Another patient had ventricular tachycardia but he refused cardioverter-defibrillator implantation and was lost to follow up. The other patient had SCD. There were no significant differences in the occurrence of significant AVB between the groups. Multivariate logistic regression analysis adjusted for age and sex showed that SQTI was independently associated with AF [odds ratio, 7.834; 95% confidence interval, 1.387–44.258; *P* = 0.020] and VA/SCA (a statistical comparison could not be performed because the incidence of VA/SCA in the normal QT group was zero) (Table 3).

**Table 2:** Clinical outcomes in the SQTI and normal QT groups

	SQTI group (n = 34)	Normal QT group (n = 136)	*P*
Arrhythmia outcomes			
AF	4 (11.8)	3 (2.2)	0.030
VA/SCA	3 (8.7)	0 (0)	0.007
Significant AVB^a^	3 (8.7)	8 (5.9)	0.461
Non-cardiac death	0 (0.0)	7 (5.1)	0.347

The results are presented as median (interquartile range) for continuous data and as frequency (percentage) for categorical data

*AF* atrial fibrillation, *AVB* atrioventricular block, *SCA* sudden cardiac arrest, *SQTI* short QT interval, *VA* ventricular arrhythmia

^a^Mobitz type 2 s-, high-, and third-degree AVB

**Table 3:** Multivariate logistic regression analysis of factors for the occurrence of AF

	Odds ratio (95% CI)	*P*
SQTI	7.834 (1.387–44.258)	0.020
Age	1.057 (1.016–1.099)	0.006
Males	0.286 (0.040–2.029)	0.211

*AF* atrial fibrillation, *SQTI* short QT interval

## Discussion

### Main findings

The main findings of the present study are as follows: (1) the prevalence of SQTI was 0.001%; (2) QT dispersion was significantly wider and heart rate was significantly slower in patients with SQTI than in those with a normal QT interval. Early repolarization, tall T waves, and U waves were more frequent in patients with SQTI than in patients with normal QT interval; and (3) SQTI was significantly associated with AF and VA/SCA.

### SQTS diagnostic criteria

Multiple studies have suggested diverse diagnostic criteria for SQTS; definite diagnostic criteria for SQTS have not been properly established since the condition was first identified. Initially, a constant QT interval ≤ 300 ms was regarded as a short QT interval.[3] After major population studies, the suggested diagnostic criteria for SQTS were a QTc ≤ 340 ms or a QTc ≤ 370 ms in the presence of a pathologic mutation; familial history of SQTS or SCA at < 40 years of age; or a history of VA in a structurally normal heart, AF in early life, or loss of consciousness that might be strongly related to a cardiac arrhythmia [6].

### Sex differences in SQTI

The majority of patients with SQTI were male and between 20 and 30 years of age. It is known that the QT interval is shorter in men than in women [7], which may be attributable to the effect of testosterone on the QTc [8, 9].

### Symptoms

Over 35% of patients with SQTI had symptoms at presentation; these included, in decreasing frequency, palpitation (n = 4, 11.8%), loss of consciousness (n = 3, 8.8%), and chest pain (n = 3, 8.8%). A previous study showed that the most frequent symptoms were palpitations and syncope, which is in accordance with the present study [10]. The cause of loss of consciousness may be self-terminating VA in patients with SQTS [11].

### ECG characteristics

A previous study demonstrated that early repolarization was associated with arrhythmias in patients with SQTS and could be used for identifying the risk of SCA in SQTS [12]. Tall T wave is one of the characteristic ECG findings in SQTS [12]. QT dispersion was significantly wider in the SQTI group than in the normal QT group in the present study. It is known that increases in repolarization dispersion are the possible substrate for reentry, which may result in VA and SCA [11]. A previous study also found that patients with SQTS had significantly wider QT dispersion [13].

### Arrhythmia outcomes

AF was the most frequent arrhythmia in the SQTI group in the present study. A prior study reported that 18.0% of patients with SQTS had AF [14]. Given the close association between SQTS and AF, the latter is included in the SQTS diagnostic criteria. This relationship was found to be attributable to a common *KCNQ1* missense mutation (*V141M*), which caused AF and shortened the QT interval by altering the gating of I_Ks_ channels [15]. Approximately 5.8% of patients experienced VA at the initial SQTI diagnosis.

### Limitations

The present study has a number of limitations. First, a small number of patients were included in the present study because SQTI is a rare condition. Large-scale prospective studies of patients with SQTI are needed. Second, genetic studies were not performed in patients with SQTI. Third, subjects with normal QT interval were recruited not from the general population but from among hospital visitors; this was decided since ECG is not routinely performed in the general population. However, subjects with normal ECG findings and without any cardiovascular disease were included to avoid selection bias. Fourth, conditions such as a history of physical activity and emotional status, which can affect QT interval, were not assessed because of the retrospective design.

## Conclusions

In this cohort of Korean population, SQTI was associated with AF and VA/SCA. It is important to note that SQTI and AF have close relationship with common *KCNQ1* mutation *(V141M)* and when SQTI is detected by taking an ECG, it is necessary to actively monitor for syncope, AF, and VA.

## Data Availability

The datasets used and analyzed during the current study are available from the corresponding author on reasonable request.

## Abbreviations

SQTS: Short QT syndrome
VA: Ventricular arrhythmia
SCA: Sudden cardiac arrest
SCD: Sudden cardiac death
ECG: Electrocardiogram
SQTI: Short QT interval
QTc: Corrected QT interval
AVB: Atrioventricular block
AF: Atrial fibrillation
